# Transcriptomic signatures of neuronal differentiation and their association with risk genes for autism spectrum and related neuropsychiatric disorders

**DOI:** 10.1038/tp.2016.119

**Published:** 2016-08-02

**Authors:** A G Chiocchetti, D Haslinger, J L Stein, L de la Torre-Ubieta, E Cocchi, T Rothämel, S Lindlar, R Waltes, S Fulda, D H Geschwind, C M Freitag

**Affiliations:** 1 Department of Child and Adolescent Psychiatry, Psychosomatics and Psychotherapy, University Hospital Frankfurt, JW Goethe University Frankfurt, Frankfurt am Main, Germany; 2 Neurogenetics Program, Department of Neurology, Center for Autism Research and Treatment, Semel Institute, David Geffen School of Medicine, University of California, Los Angeles, Los Angeles, CA, USA; 3 Department of Biomedical and NeuroMotor Sciences, University of Bologna, Bologna, Italy; 4 Institute of Legal Medicine, Hannover Medical School, Hannover, Germany; 5 Institute of Experimental Cancer Research in Pediatrics, Frankfurt am Main, Germany

## Abstract

Genes for autism spectrum disorders (ASDs) are also implicated in fragile X syndrome (FXS), intellectual disabilities (ID) or schizophrenia (SCZ), and converge on neuronal function and differentiation. The SH-SY5Y neuroblastoma cell line, the most widely used system to study neurodevelopment, is currently discussed for its applicability to model cortical development. We implemented an optimal neuronal differentiation protocol of this system and evaluated neurodevelopment at the transcriptomic level using the CoNTeXT framework, a machine-learning algorithm based on human post-mortem brain data estimating developmental stage and regional identity of transcriptomic signatures. Our improved model in contrast to currently used SH-SY5Y models does capture early neurodevelopmental processes with high fidelity. We applied regression modelling, dynamic time warping analysis, parallel independent component analysis and weighted gene co-expression network analysis to identify activated gene sets and networks. Finally, we tested and compared these sets for enrichment of risk genes for neuropsychiatric disorders. We confirm a significant overlap of genes implicated in ASD with FXS, ID and SCZ. However, counterintuitive to this observation, we report that risk genes affect pathways specific for each disorder during early neurodevelopment. Genes implicated in ASD, ID, FXS and SCZ were enriched among the positive regulators, but only ID-implicated genes were also negative regulators of neuronal differentiation. ASD and ID genes were involved in dendritic branching modules, but only ASD risk genes were implicated in histone modification or axonal guidance. Only ID genes were over-represented among cell cycle modules. We conclude that the underlying signatures are disorder-specific and that the shared genetic architecture results in overlaps across disorders such as ID in ASD. Thus, adding developmental network context to genetic analyses will aid differentiating the pathophysiology of neuropsychiatric disorders.

## Introduction

Autism spectrum disorders (ASD) share some of their genetic architecture with other neuropsychiatric disorders such as fragile X syndrome (FXS), schizophrenia (SCZ) and intellectual disabilities (ID). It is known that the fragile X mental retardation protein (FMRP), a translational repressor causal for FXS, specifically binds ASD risk genes,^1^ and common variants of genes involved in the regulation of FMRP pathways are associated with ASD.^2^ In addition, several genes affected by copy number variations (CNVs) and mutations detected in ASD patients were also associated with SCZ^3, 4^ or ID.^5, 6^ However, integrative analyses investigating the functional overlap between ID and ASD showed that only ASD genes, but not ID-implicated genes, are enriched in neocortical developmental networks.^7, 8^ Thus, it is unclear to which extent the genetic overlap reflects shared or differential pathomechanisms. Investigating the effect of disease risk genes on neurodevelopment is of major interest to understand the etiology and pathophysiology of these neuropsychiatric disorders. Feasible, reproducible and scalable cell models are thus needed to elucidate the functional consequences of genetic variants. Human neuronal models used so far include neurons differentiated from embryonic stem cells, induced pluripotent stem cells (iPSCs), human neuronal progenitor cells (NPCs) or neuroblastoma cell lines. The choice between the different cell models involves clear tradeoffs. The SH-SY5Y neuroblastoma cell line is the most cited *in vitro* model in neuropsychiatric research and has the advantage of low cost, ease of culture (feasibility), reproducibility and available literature. Despite its origin from a tumour, its neuroectodermal lineage allows investigating neuronal phenotypes of neurodevelopmental and neurodegenerative diseases. New models based on embryonic stem cells or iPSCs promise to recapitulate *in vivo* development more faithfully than this or other cell lines,^9^ leading to the question of the most appropriate use of SH-SY5Y models in modelling of neuropsychiatric disorders. Yet, it should be acknowledged that embryonic stem cell, iPSC and human NPC models are expensive, and culturing and differentiating of these cells in a reproducible manner is far more difficult than for SH-SY5Y cells.^10, 11^ Recognising the limitations of all model systems, it therefore is important to understand how the optimal protocol for using the efficient SH-SY5Y model system recapitulates *in vivo* development, which will be helpful in guiding this and future studies. In summary, we focused on improving neuronal differentiation of SH-SY5Y cells because of feasibility, reproducibility, the broad use of this model in previous publications and the large data resources available.

Goldie *et al.*^12^ summarised that 72% of SH-SY5Y differentiation protocols used all-trans retinoic acid (RA) only, whereas others used a sequential treatment with RA and brain-derived neurotrophic factor (BDNF) or alternative agents such as 12-*O*-tetradecanoylphorbol-13-acetate. However, no study has used RA and BDNF in combination, as it is currently used in NPC and iPSC differentiation protocols. BDNF is strongly involved in differentiation and maintenance of cortical neurons.^13, 14^ Here, we tested whether a continuous exposure of SH-SY5Y to RA and BDNF (cRA-BDNF) improves differentiation ability compared with RA only or subsequent RA-BDNF (sRA-BDNF) protocols. Therefore, we evaluated publicly available and newly generated transcriptomic signatures of these three methods (RA-only;^15^ sRA-BDNF;^16^ and cRA-BDNF, the data set produced here) for their capacity to model cortical development implementing the CoNTeXT framework^9^ specifically developed for this use. The CoNTeXT framework is based on a machine-learning algorithm trained on genome-wide transcriptomic data available through the Brain Span Atlas of the developing brain (www.brainspan.org). This data set includes cross-sectional transcriptomic data across all human brain regions spanning the whole lifespan (2 weeks after conception to > 60 years^17, 18^). The CoNTeXT framework predicts developmental stage and region of origin of a transcriptomic signature, analyses gene-network preservation based on weighted gene co-expression analysis and compares developmental transitions. The accuracy of predictions can be estimated based on empirical modelling using rank–rank hypergeometric overlap (RRHO) tests provided in the original publication by Stein *et al.*^9^

To then exploit our improved cell system, we performed a biostatistical analysis of generated transcriptomic signatures. Finally, we delineated specific regulatory modules and signatures of neuronal differentiation and their association with neuropsychiatric disorders comparing ASD with FXS, ID and SCZ.

## Materials and methods

### Neuronal differentiation of SH-SY5Y

SH-SY5Y identity was confirmed by DNA fingerprinting using AmpFℓSTR Profiler and AmpFℓSTR NGM Select PCR Amplification Kits (Applied Biosystems, Foster City, CA, USA) according to the manufacturer's protocol. Cells were proliferated under standard conditions (Dulbecco's modified Eagle medium, 10% fetal bovine serum, 1% sodium pyruvate (all Life Technologies, Carlsbad, CA, USA) and 1% penicillin/streptomycin (PAA, Dartmouth, MA, USA)). Here, we used a protocol for neuronal differentiation applying RA and BDNF simultaneously in contrast to currently used standard protocols.^19^ Cells were differentiated in Neurobasal-A medium with 1 × GlutaMAX, 1 × B-27 supplement (all Life Technologies), 10 μM RA, 2 mM cAMP (both Sigma-Aldrich, St. Louis, MO, USA), 50 ng ml^−1^ hBDNF (Immunotools, Altenoythe, Germany), 1% PAA and 20 mM KCl over a time course of 11 days changing the medium every other day. The time points for mRNA analysis were set 24 h after changing the medium and were as follows: 0 (undifferentiated cells), 1, 3, 5, 7, 9 and 11 days *in vitro* (DIV).

#### Validation of differentiation

Markers for neuronal differentiation were analysed at mRNA and protein levels using real-time reverse transcriptase-PCR (RT-PCR) and western blot analyses, respectively. We used *CDK1* as marker for cell division and *MAPT* as marker for axonal outgrowth (Supplementary Methods and Supplementary Table 1).

### Whole-genome expression analysis

#### mRNA microarray raw-data SH-SY5Y

For whole-transcriptome analysis of the SH-SY5Y cell line, whole RNA of three biological replicates of time points 0 DIV to 11 DIV was extracted using the GeneJet RNA Purification Kit according to the manufacturer's protocol (Fermentas) including DNase treatment. All samples passed quality check analysis (RNA Integrity Number RIN>7, Bioanalyzer, Agilent, Santa Clara, CA, USA). Microarray analysis using HumanHT-12 v4 Expression BeadChips (Illumina, San Diego, CA, USA) was outsourced to Atlas Biolabs (Berlin, Germany). Raw intensity data for each probe were extracted using GenomeStudio software v2011.1 (Illumina). The mean signal intensities per probe were exported for further analysis. Microarray data can be accessed through the gene expression omnibus repository (GEO) under the accession number GSE69838.

#### Computational analysis

All analyses were conducted in R-program version 3.0.2., if not otherwise specified.

Pre-processing and quality check analysis: Microarray raw data were log2-transformed and quantile-normalised. All Illumina probe IDs were matched to the respective annotated Entrez Gene ID using the '*biomaRt'* Package. Entrez Gene IDs were mapped to the official human genome nomenclature symbol. If genes were targeted by more than one probes, the probe with the highest variance was chosen for further investigation. A total of *N*=20 318 unique annotated genes were targeted by the microarray (Supplementary Table 2). Genes that were significantly (detection *P*-value<0.05) expressed above background in all replicates were defined as the expressed neuronal transcriptome (*N*=11 392).

Hierarchical cluster analysis of the top 2 000 genes by variance (distance=1−Pearson correlation, method=average linkage) was used to identify methodological outliers. No samples presented with high branch points in the cluster dendrogram. Principal component analysis confirmed similarity of the biological replicates (Supplementary Figure 3).

Microarray mRNA expression of selected ASD-risk genes and neuronal differentiation-specific markers were validated using relative real-time RT-PCR and compared using Pearson correlation analysis (Supplementary Methods, Supplementary Figure 1 and Supplementary Table 1).

CoNTExT analysis: We used the recently developed framework of algorithms matching *in vitro* mRNA expression signatures to transcriptomic atlases of *in vivo* brain development. The CoNTExT algorithm (http://context.semel.ucla.edu/) is a machine-learning framework that determines the temporal and regional identity as well as preservation of co-regulated modules based on expression signatures from 18 different stages of the human lifespan representing early embryonic stage to an age greater than 60 years. Accuracy is dependent on the overlap in gene signatures between the *in vitro* and the *in vivo* data. Given a −log10(*P*-value)=299 in the RRHO analysis, the accuracy of predictions was estimated to be >96%. The effect of time on CoNTeXT scores for each stage and region and for each data set were tested using linear regression models. Details on the algorithms and accuracy estimation are published elsewhere.^9^

Data sets used for comparison of SH-SY5Y differentiation protocols: Raw data were downloaded from the gene expression omnibus GEO database (http://www.ncbi.nlm.nih.gov/geo/). The RA-only data set (Korecka data set) included transcription signatures of cells cultured for 8 days with and without all-trans RA only;^15^ GEO Acc Nr: GSE43368. The sRA-BDNF data set (Nishida data set) included the ECACC-derived SH-SY5Y cell line treated with RA and BDNF for 5 and 3 days subsequently;^16^ GEO Acc Nr: GSE9169. All data sets were log2-transformed and quantile-normalised as described above.

Identification of differentially expressed genes: In this analysis only the expressed gene set (*N*=11 392) was used. We applied linear fixed-effect models ('*nlme'* package in R) accounting for random effects between the three replicates with the two dichotomized time points (for example, 0 DIV vs 3 DIV) predicting expression values. Only differentially expressed (DEX) genes with (Benjamini–Hochberg) false discovery rate (FDR)<0.05 (accounting for *N*=11 392 tests) were included in GO term analysis.

Dynamic time warp analysis: Wexler *et al.*^20^ have successfully adapted Dynamic time warp (DTW) analysis to identify time-dependent gene activation. The authors provided evidence that the DTW distance increases linearly with background noise. DTW analysis was performed using the ‘*dtw*' package in R. All expressed genes were analysed (*N*=11 392) and compared with a noise matrix (Supplementary Methods). We defined the maximum DTW distance (DTW dissimilarity score) as the intercept of a linear model predicting the DTW distance in dependence of background noise for each gene in our cell line model (Supplementary Figure 4).

Genes with a DTW distance larger than 2 × noise intercept (upper 95% boundary) were considered for further analysis. Genes with similar regulatory patterns were grouped by k-means cluster analysis on the Euclidean distances. Optimal number of clusters was defined applying the ‘elbow criterion' based on the Scree-plot of within-cluster sum of squares around cluster means (Supplementary Figure 4) as suggested.^21^

Parallel independent component analysis: Parallel independent component analysis (pICA) analysis is an exploratory approach based on blind source separation techniques to identify regulatory genes contributing to biological processes.^20, 22^ Here, we used the ‘*fastICA'* package in R-program and iterated the *pICA* 250 times extracting two to eight components, respectively (Supplementary Methods). A total of four components was extracted with a mean correlation coefficient *r*>0.999 of all gene loads (first principle component) over all iterations. Genes were considered as significant if their load within the components was above the conservative cutoff^20^ of score >3 (gene load; Supplementary Figure 5). The analysis was performed using the whole neuronal transcriptome (*N*=11 392).

Weighted gene co-expression network analysis: Weighted gene co-expression network analysis (WGCNA) analyzes co-expression patterns between genes and identifies gene sets (modules) based on their topological overlap (Supplementary Methods). WGCNA was performed using the ‘WGCNA' package in R as recommended.^23^ Here, to identify network modules of regulatory genes only, we used the neuronal transcriptome gene set (*N*=11 392) and calculated a topological overlap matrix based on a signed adjacency (Supplementary Methods and Supplementary Figure 6).

Network analysis and hub gene identification: Genes with a high functional impact within a network are hypothesized to be highly connected and members of several regulatory pathways (that is, hub genes). The connectivity of a gene is defined by the sum of its weighted correlation with the other genes as defined through the adjacency measure in the WGCNA. We also defined network centrality (Degree) of a node (number of connections a gene has) and Betweenness (that is, the number of shortest connections across all nodes passing through the node of interest). To reduce hyperconnectivity, we only considered two genes to be connected (Degree) if their expression correlated with a Spearman cor>0.90. All calculations were performed using the ‘*igraph*' package.

GO-term enrichment analysis: GO-term enrichment was performed with the ‘topGO' package using the *weight01* algorithm, where the significance of a GO-term is weighted by the enrichment score of related GO terms in combination with a bottom-up elimination algorithm,^24^ increasing the efficiency in detecting relevant associated terms. Individual tests are not independent, and therefore multiple testing does not apply. *P*-values extracted thus can be considered as corrected for multiple testing. The gene universe was defined as the 11 392 genes significantly expressed.

Risk gene enrichment analysis: In total, we tested the following 14 gene lists: gene counts refer to genes targeted on our chip:*' 01_AutismKB':* genes (*N*=3 050), non-syndromic and/or syndromic, listed in AutismKB; ‘*02_AutismKB_core*': genes (*N*=170) included in the high reliability core data set of the AutismKB database (for details on these two lists see http://autismkb.cbi.pku.edu.cn).^25^ *‘03_SFARI_all':* all 341 genes listed in the SFARI Gene Autism database,^26^ excluding non-supported genes. *‘04_SFARI_score4plus':* all 250 genes listed in the SFARI Gene Autism Database with a score ⩾4, that is, genes with minimal evidence in at least one candidate gene study. *‘05_Voineagu_M12':* *N*=443 genes within a network module (M12) identified to be differentially regulated in post-mortem brains of ASD individuals.^27^ *‘06_Voineagu_M16':* genes (*N*=386) also reported by Voineagu *et al.*^27^ *‘07_Gilman_Netbag': ASD-risk* genes (*N*=72) identified through a network-based analysis of ASD associated rare *de novo* CNVs.^28^ *‘08_Iossifov_RDNV':* genes (*N*=672) targeted by rare *de novo* likely gene-disrupting variants identified through exome sequencing.^29^ *‘09_DeRubeis_RCV':* genes (*N*=100) recurrently targeted by rare coding variants.^30^ ‘*10_Darnell_FMRP*': genes (*N*=837) whose mRNAs are directly bound by the FMRP gene.^1^ ‘*11_Parikshak_ID_genes':* genes (*N*=388) implicated in ID retrieved from multiple publications.^7^ *‘12_Pinto_ID_genes':* manually curated list of genes (*N*=252) implicated in monogenic ID.^5^ *‘13_Fromer_SCZ_allRNDV':* genes (*N*=676) targeted by rare *de novo* variation in SCZ.^31^ *‘14_Cocchi_Pruning':* a manually curated list of (*N*=117) genes involved in pruning.^32^ For detailed information on all gene lists see Supplementary Table 3 and Supplementary Figure 7).

Enrichment testing was performed applying Fisher's exact test. Benjamini–Hochberg correction for multiple testing (number of risk-gene lists times number of clusters identified in each analysis) was applied. As the reference genome, the list of all 20 318 Entrez Genes targeted on the chip was used.

## Results

### Basic confirmation of neuronal differentiation

SH-SY5Y cells continuously exposed to RA and BDNF (cRA-BDNF) for 11 days yielded a stereotypical multipolar neuronal morphology with one to two long axon-like processes and several shorter dendrite-like processes as reported in the original publication by Encinas *et al.*^19^ Robust differentiation was confirmed by known markers for cell cycle (*CDK1*) and axonal outgrowth (*MAPT*), which were down-regulated and up-regulated, respectively (Supplementary Figure 1a). Microarray expression data of ASD risk genes (*SHANK3*, *NRXN1*, *CNTNAP2*, *DHCR7*, *GABRB3* and *GRIK2)*, glutamatergic receptors (*GRIN1*, *GRIA2*, *GRM1* and *GRM4)* and dopaminergic markers (*TH* and *DRD4*) that were previously reported to be regulated during neuronal differentiation,^33^ showed high correlation with expression levels assessed by real-time RT-PCR demonstrating technical reproducibility of microarray data (correlation coefficients ranging from 0.561 *(GRIA2*) to 0.984 (*NRXN1*); Supplementary Figure 1b). Markers for neuronal subtypes, including cholinergic, dopaminergic, serotonergic, GABAergic and glutamatergic neurons were expressed (Supplementary Figure 2). The dopamine transporter (*DAT1*) was strongest expressed at undifferentiated stages and down-regulated during differentiation. The dopaminergic marker TH (Supplementary Figure 2) was not regulated, whereas cholinergic markers (*ACHE*, *SLC18A3*) were up-regulated. We also observed a modest up-regulation of glutamatergic (*SLC17A7*) and GABAergic (*SLC32A1*) transporters. Markers specific for motor neurons were expressed but not regulated during differentiation. The implemented differentiation protocol thus yielded an unspecific mixture of neurons.

### Evaluation and comparison of the improved neuronal differentiation method

Similar to findings in the human brain,^34^ the SH-SY5Y neuronal transcriptome, that is, the genes expressed above microarray background in our cell model included 11 392 out of 20 318 genes targeted on the microarray.

**Figure 1:**
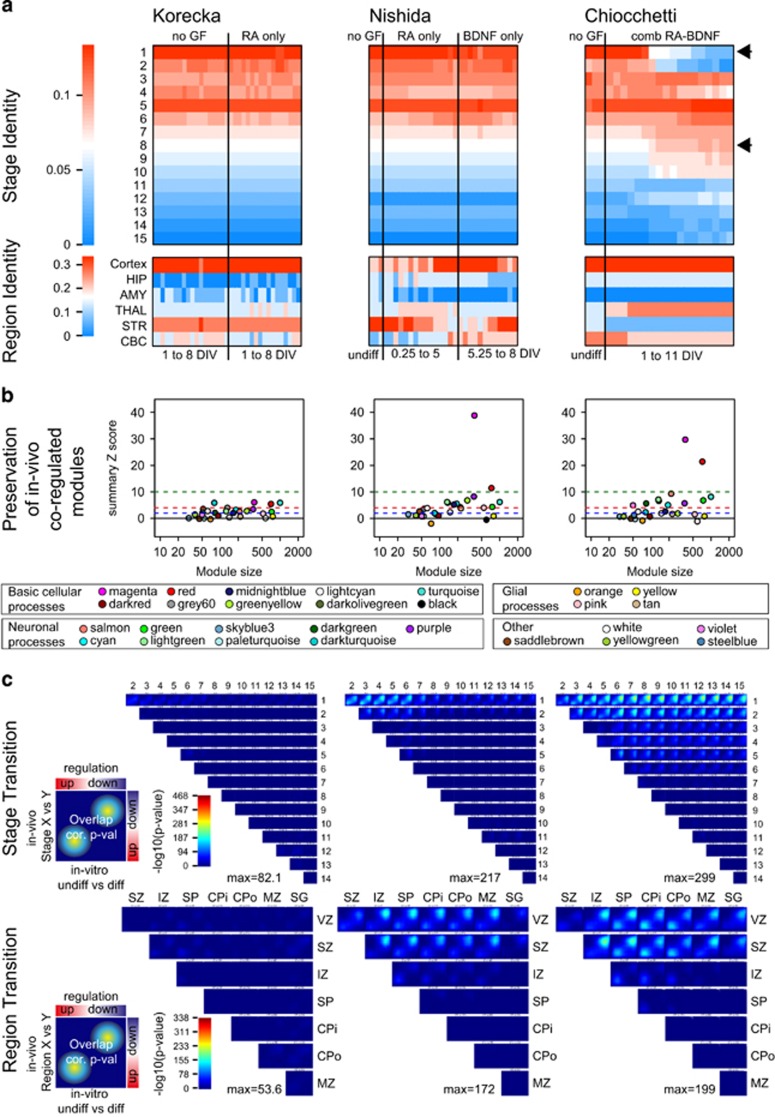
Evaluation of neuronal differentiation comparing three SH-SY5Y protocols. (**a**) CoNTExT analysis comparing mRNA signatures previously published (Korecka; Nishida) and generated here (Chiocchetti). mRNA signature of the cells (Chiocchetti data set) differentiated by continuous exposure to retinoic acid (RA) and brain-derived neurotrophic factor (BDNF) was showing a more mature phenotype than the other two sets. Our data set was most similar to the cortical area and was reminiscent of 15–19 weeks post conception (Stage 5) or above. In addition, our data set showed no reminiscence for earlier stages after 11 days *in vitro* (DIV; see black arrows). Regression analyses testing association of time with CoNTeXT scores are provided in Supplementary Table 4. (**b**) Module preservation analysis comparing co-expression network modules *in vivo* vs *in vitro*. *Z*-scores are plotted against the number of genes within each module. Highest significant preservation (*Z*-score) is observed for cell cycle-related (red, magenta) modules in both data sets using BDNF (Nishida and Chiocchetti). In our set-up, neuronal function modules such as ‘glutamatergic synaptic transmission, axon and dendrite development' (salmon, green) or ‘GABAergic synaptic transmission and synaptic vesicle exocytosis' (lightgreen) were nominally significant, whereas ‘axon guidance, neuronal migration and GTPase activity' (purple) show intermediate (*Z*-score 2–10) preservation. No preservation was observed for modules related to synaptic transmission (pale-turquoise, yellow), gliogenesis (tan, yellow) or immune response (black, orange) in any data set. Dashed lines mark *Z*-scores blue=1.96, red=4, green=10. For details see Supplementary Table 4. (**c**) Mapping of transitions between stages and cortical layers is shown as rank–rank hypergeometric overlap (RRHO) maps. Genes were ranked based on signed *P*-values comparing undifferentiated versus differentiated cells. These ranks were binned (200 genes each) and respective genes were tested using a hypergeometric test for overlap with the genes in all bins generated of *in vivo* comparison of stages (*x* vs *y* axis) or cortical layers, respectively. *P*-values of each comparison are plotted. The coloring of −log10 (*P*-values) is scaled to the maximum values observed in the original publication by Stein *et al.*^9^ The maximum *P*-value within each analysis is shown as a measure for overall accuracy. Simulations performed by Stein *et al.*^9^ predict that given a –log10(*P*-value)~300 in the stage transition mapping the CoNTeXT algorithm can predict the developmental stage with an estimated accuracy ~96% and brain region accuracy ~90%. For details see original publication.^9^ AMY, amygdala; CBC, cerebellar cortex; HIP, hippocampus; STR, striatum; THAL, thalamus.

The CoNTExT framework^9^ was used to estimate differentiation stage and brain-regional identity of our cell model. Expression signature of cRA+BDNF SH-SY5Y cells was reminiscent of brain tissue developed for at least 15–19 weeks post conception (Stages 5–8; accuracy>96%) and was most likely to be of a cortical identity (accuracy>90% Figure 1a). In contrast, the two published data sets on SH-SY5Y neurodevelopment that differentiated cells either by RA treatment-only (RA-only)^15^ or by sRA-BDNF^16^ did not show a transcriptional phenotype as mature as cells differentiated by the continuous exposure to RA and BDNF as used here. Regression analysis of CoNTeXT scores confirmed this observation: changes in our data set for stages 1, 2, 4–10, 12 and 15 were significantly depending on time (Supplementary Table 4), whereas no significant association between differentiation over time and CoNTeXT scores was identified for the Korecka data set. Only scores for Stage 1 of sRA-BDNF-treated SH-SY5Y were significantly altering over time. In summary, RA-only cells were least mature, followed by sRA-BDNF cells.

cRA+BDNF and sRA-BDNF cells matched cortical regions with no significant changes over time, whereas RA-only were striatal at the beginning of differentiation and turned more cortical at later stages. However, changes were not significant. Co-regulatory network modules (Figure 1b) were specifically preserved in the sRA-BDNF and cRA+BDNF, but not the RA-only-treated cells. This included preservation of mitosis, neuronal development, glutamatergic and GABAergic transmission, but not modules related to synaptogenesis, glia-genesis or immune response (for details see Supplementary Table 4 and previous publications by Stein *et al.*^9^). In the RRHO maps (Figure 1c), only our improved model showed similarities with the transition from early stages 1 or 2 to stage 5 and beyond, including both down and up-regulated gene sets. Mapping of transcriptional signatures of mid-fetal cortical layers from gestational stage 5–6 (ref. 18) revealed that sRA-BDNF and cRA+BDNF treatments can model the transition of cells from the ventricular layers to the subplate layer, where the cRA+BDNF system showed highest significance and accuracy of prediction (96% for stage and above 85% for regional identity; for details see Materials and Methods section and original CoNTeXT publication^9^).

### Identification of genes implicated in neuronal differentiation

**Figure 2:**
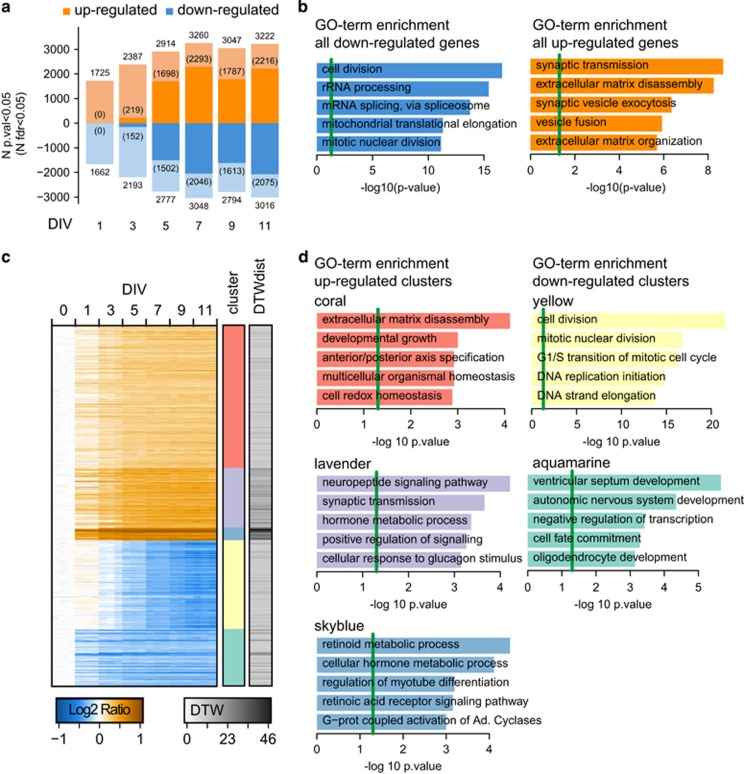
Genes differentially expressed (DEX) over time during differentiation. (**a**) Number of genes significantly DEX comparing each time point (each *N*=3) to 0 DIV (day *in vitro*; N=3). Most genes are DEX after 7 DIV. Number of genes with an false discovery rate (FDR)<0.05 are shown in parentheses in a darker shade. No DEX gene at 1 DIV survived correction for multiple testing. (**b**) GO-term enrichment of DEX genes. Neuron-related biological processes including synaptic transmission or vesicle exocytosis are enriched in up-regulated gene sets; cell division and translation-related processes in the down-regulated set, respectively. Weighted *P*-values of the five most significant terms are shown. Green line marks the weighted *P*<0.05 cutoff. (**c**) Heatmap of 509 genes identified to be dynamically regulated over time (dynamic time warp (DTW) analysis). Genes were selected based on their DTW distance (DTWdist) score (grey values) when compared with the expected background noise signal calculated for each gene (Supplementary Figure 4). On the basis of k-means cluster analysis and this heatmap, we decided to cluster genes into five potential regulatory groups. (**d**) GO-term enrichment analysis of identified clusters shows up-regulated clusters to be enriched for developmental processes (coral), neuronal signaling (lavender) and retinoic acid metabolism (skyblue). Down-regulated clusters are related to cell cycle regulation (yellow) and cellular development and fate (aquamarine). Weighted *P*-values of the five most significant terms are shown. Green line marks the weighted *P*<0.05 cutoff.

To robustly identify the subset of genes regulated during neural development, we implemented three complementary statistical approaches: DEX analysis identifies genes by comparing two time points, DTW analysis identifies genes that are regulated across a temporal trajectory and ICA extracts individual processes and underlying genes. Similar to a recent publication,^35^ a total of *N*=6 049 genes were DEX with a FDR<0.05 at least at one time point compared with undifferentiated cells (Figure 2a and Supplementary Table 5). *N*=3 169 genes were up-regulated and *N*=2 894 genes down-regulated, of which 14 genes were up-regulated at one time point and down-regulated at another (*ALDH9A1, BCL11A, CNDP2, CTSF, EPHB4, GREM2, KCNG1, MRAP2, NET1, PHKB, SCAND1, TEAD2, TRAM2* and *ZNF217*). No gene was significantly (FDR<0.05) regulated after 1 day of differentiation. However, a total of 3 387 genes were regulated with nominal significance at that time point. Most of the transcriptional regulation occurred during the first week. In accordance, we observed higher similarities between later time points than earlier time points (Supplementary Figure 3). GO-term analysis attributed up-regulated DEX genes to synaptic exocytosis and transmission and extracellular matrix re-organization, whereas genes down-regulated belonged to GO-terms mitosis or DNA replication (all weighted *P*-values <10E−4; Figure 2b and Supplementary Table 5). A similar pattern was observed when analyzing regulated genes of each time point individually, linearly with the expected induction of neuronal differentiation and the cessation of cell division.

The DTW approach identified 509 genes with continuous or dynamic changes over time (Figure 2c, Supplementary Table 6 and Supplementary Figure 4). All but four of these genes were a subset of the DEX gene set. Consequently, 8.3% of the total DEX genes were also dynamically regulated during neuronal differentiation.

The DTW genes clustered into five trajectories or stimulatory responses (Figure 2c); three showed a positive response (coral, lavender and skyblue clusters) and two a negative response (yellow and aquamarine clusters). The cluster with the strongest positive regulation (skyblue) comprised 18 genes significantly enriched for RA response (Figure 2c and Supplementary Table 6). The coral (low-positive responder) and lavender (intermediate-positive responder) were enriched for genes related to developmental processes (GO-terms extracellular matrix disassembly and developmental growth) and neuronal signaling, respectively. The down-regulated clusters were associated with cell division and cell cycle transition (yellow) or autonomic nervous system development and ventricular septum development (aquamarine). The yellow cell cycle cluster showed an initial weak positive response after induction of differentiation before changing to the inhibitory response.

**Figure 3:**
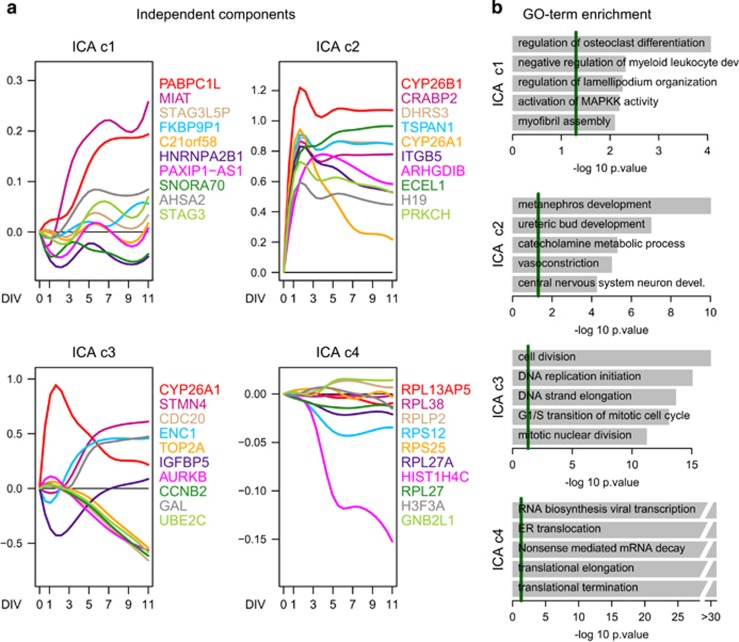
Parallel independent component analysis (pICA) of gene expression. (**a**) Log2 fold-change of signal intensities normalised to time point 0 DIV of the top 10 genes within the four reproducible extracted components. (**b**) GO-term gene set enrichment analysis of the individual components. Weighted *P*-values of the five most significant terms are shown. Green line marks the weighted *P*<0.05 cutoff.

Finally, four components were observed by ICA (Figure 3a) with a total of *N*=699 high loading genes (Supplementary Table 7 and Supplementary Figure 5). On the basis of GO-term enrichment analysis (Figure 3b and Supplementary Table 7), the four biological entities (C1–4) were labeled as differentiation and cellular organization (C1), developmental processes (C2), cell cycle regulation (C3) and protein synthesis and modification processes (C4). In addition, C1 contained several zinc-finger proteins and DNA-binding proteins supporting the idea that C1 is a transcriptional regulatory process.

**Figure 4:**
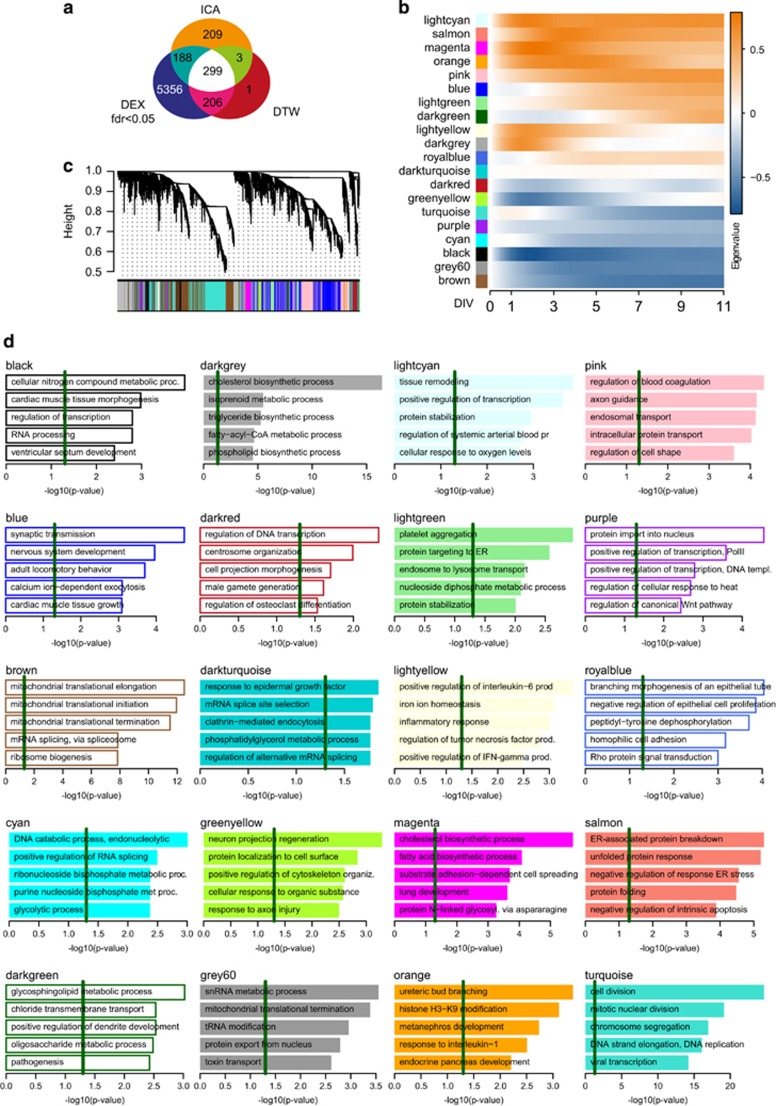
Overlap of results and weighted gene co-expression network analysis. (**a**) Overlap of gene sets identified through linear regression (false discovery rate (FDR)<0.05; Figure 2a) DEX, dynamic time warping analysis (DTW; Figure 2c) and parallel independent component analysis (pICA; Figure 3). (**b**) Weighted gene co-expression network analysis (WGCNA) module definition based on topological overlap; colors correspond to modules identified. (**c**) Module Eigenvalues (that is, first principal component; Eigengene) of log2 fold changes of the respective modules normalised to time point 0 DIV. (**d**) GO-term enrichment analysis of identified network modules. Weighted *P*-values of the five most significant terms are shown. Green line marks the weighted *P*<0.05 cutoff.

Combining all genes identified through DEX, DTW and ICA analyses yielded 6 262 genes implicated in neuronal differentiation. Of these, 299 were identified in all three analyses (Figure 4a). This high-confidence gene set was implicated in cell cycle regulation.

### Co-expression network analysis

WGCNA identified 20 regulatory modules (Figures 4b–d and Supplementary Table 8). As expected, modules up-regulated early during neuronal differentiation were enriched for processes needed for membrane remodeling (magenta), protein stabilization (lightcyan) or axonal guidance (pink). Late activated modules were associated with synaptic transmission (blue) or dendrite development (darkgreen). Modules up-regulated during early phases of differentiation only were related to inflammatory response (lightyellow) and cellular fatty-acid metabolism (darkgrey).

In contrast, early down-regulated modules were attributed to cell division (black) or mitochondrial organization (grey60, brown). Modules inhibited at later stages also included genes associated with cell cycle regulation (turquoise) and DNA metabolic processes (cyan, purple). Modules associated with splicing (darkturquoise) or cell projection and transcriptional regulation were undulating over time.

### Enrichment of risk genes in regulatory gene modules of neuronal differentiation

**Figure 5:**
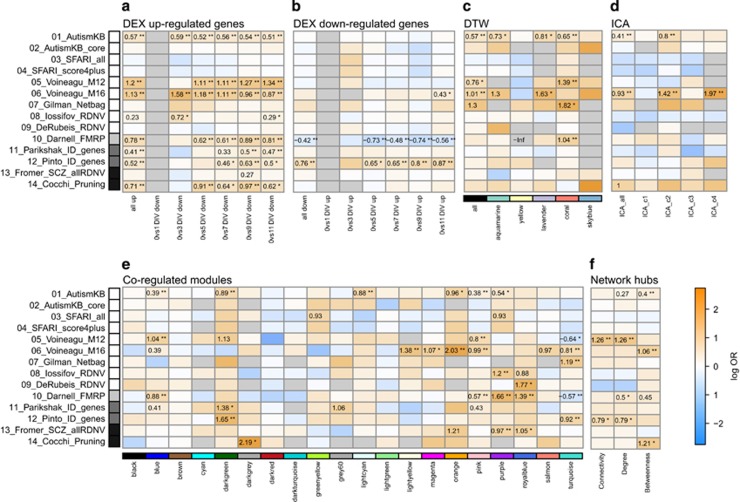
Risk gene enrichment analysis. Disorder-implicated risk-gene lists were tested for enrichment in (**a**) up-regulated differentially expressed genes (DEX), (**b**) down-regulated DEX genes (no gene survived correction for multiple testing comparing time point 1 DIV; thus, enrichment testing was not applicable), (**c**) genes dynamically regulated (dynamic time warp (DTW)), (**d**) genes contributing to biological regulatory components (ICA); **e**) modules identified by the weighted gene co-expression network analysis (WGCNA) and (**f**) the top 10% of genes based on Connectivity, Degree and Betweenness. Connectivity is the sum of all adjacencies for a given gene. Degree is defined as the number of connections and Betweenness centrality is the number of shortest paths in a network passing through a given gene. For details on the tested gene lists, see Materials and Methods section. Log-transformed odds ratios are shown if the respective false discovery rate (FDR)<0.1. Asterisks mark significance: *FDR<0.05; **FDR<0.01. Shaded boxes on the left of heatmaps correspond to genes implicated in autism spectrum disorder (ASD; white), fragile X syndrome (FXS; light grey), intellectual disabilities (ID; dark grey) and schizophrenia (SCZ; black).

We next tested identified modules and gene sets for enrichment with genes implicated in either ASD, FXS, SZ or ID using 14 published lists of disorder-specific risk genes (Supplementary Table 5). We first tested the up- and down-regulated DEX gene sets and observed a significant (FDR<0.05) enrichment among the combination of all genes up-regulated at any time point for risk genes of all four disorders (Figures 5a and b and Supplementary Table 9). The strongest enrichment (log odds ratio (OR)=1.20, FDR<0.01) was observed for genes belonging to a network module (M12) that has previously been identified to be DEX in ASD brains.^36^ Among the set of down-regulated genes, only genes implicated in ID, but not in any of the other neuropsychiatric disorders, were significantly enriched. When studying the individual time points, enrichment for ASD risk genes listed in the Autism knowledgebase (AutismKB) was similar over all time points. However, genes up-regulated at later time points (7–11 DIV) were more likely (log OR>0.81, all FDR<0.01) to be FMRP targets than those at earlier time points (2 DIV: log OR=0.32, FDR>0.1; 5 DIV: log OR=0.61, FDR<0.01). In addition, we observed significant under-representation (log OR <−0.73, FDR<0.01) after 5 and 9 DIV of these targets among down-regulated genes (Figures 5a and b). Genes regulated after 5, 7 and 11 DIV were enriched for ID-implicated genes only.

In addition, enrichment for the ASD-associated module M12, which is a synapse and neuron function-related gene cluster, increased over time, whereas enrichment for the glia and ASD-associated module M16 decreased. SCZ-implicated genes targeted by *de novo* mutations showed a weak association (FDR<0.1) with up-regulated genes after 7 DIV. Genes implicated in synaptic pruning were, similar to ASD genes, enriched among up-regulated genes only.

Next, we investigated whether the risk genes implicated in the neuropsychiatric disorders of interest here were enriched in dynamically regulated genes (DTW genes; Figure 5c). We report an association with ASD-risk genes, specifically when testing all DTW genes (log OR=0.57, FDR<0.01). FMRP-targeted genes were enriched among the set of genes associated with extracellular matrix assembly and developmental growth. ASD genes listed in the AutismKB database were enriched among the aquamarine (nervous system development), the lavender (synaptic transmission) and the coral (development) clusters. No significant enrichment was identified for SCZ- or ID-implicated genes.

When testing the specific underlying biological processes (that is, independent components; ICA), only genes listed in AutismKB and the glia-associated ASD-module M16 were found to be enriched among genes contributing to developmental processes (ICA c2) or translational processes (ICA c4; Figures 5d).

ASD genes were predominately enriched among up-regulated modules identified through WGCNA, that is, the blue, darkgreen, lightcyan, orange and pink modules (Figure 5e). These modules refer to synaptic transmission, dendrite development, tissue remodeling, histone modifications and axon guidance, respectively. The blue and pink modules were also associated with FMRP targets. One down-regulated module (purple, transcriptional response to stress) and one up-regulated gene set (royalblue, branching, cell adhesion and morphological development) were associated with genes identified in mutation screens of ASD and SCZ as well as FMRP targets (all log OR>0.97, FDR<0.05), but not with ID-implicated genes. Despite the overlap of risk genes between ASD and ID, only the darkgreen module was associated with ID and ASD. Again, for ID genes we observed that the down-regulated darkturquoise gene set is significantly enriched for ID genes but not for FMRP targets.

Finally, we constructed a correlation network of the neuronal transcriptome to test the hypothesis that genes implicated in neuropsychiatric disorders are likely to be among the top connected hub genes, defined as the upper 10% using network measures for Connectivity, Betweenness and Degree (for details see Materials and Methods). Here, we showed that ASD and ID genes are enriched among two different types of hub genes: ID genes can be found among highly connected genes (Degree, Connectivity; log OR=0.79, FDR<0.05), whereas ASD risk genes were among hubs with high influence on information transfer within a system (Betweenness, log OR=0.40, FDR<0.01), that is, genes that potentially connect networks or modules (Figure 5f and Supplementary Table 9).

Voineagu's Module M16 genes (log OR=1.06, FDR<0.01) and pruning genes (log OR=1.21, FDR<0.01) were also enriched among highly connected genes, whereas genes belonging to gene set M12 were likely to be among information transfer genes (Betweenness, log OR=1.06, FDR<0.01).

## Discussion

### Continuous exposure to RA and BDNF of SH-SY5Y cell line improves neuronal differentiation

As the utility of the SH-SY5Y model to study neuronal differentiation depends at least partially on how well it models *in vivo* development,^9^ we tried to improve current differentiation protocols and evaluated the capacity of these differentiated cells to model cortical development. One major limitation of this model is its origin from a tumor. The cell line shows several well-described cytogenetic aberrations including oncogene-spanning CNVs.^37^ Tumor-derived cell lines have a highly active, dysregulated cell cycle, which might bias the interpretability of cell cycle-associated findings. However, to our knowledge, the reported CNVs do not span major genes associated with neuronal differentiation and neuropsychiatric disorders under study here.

The result of the CoNTExT algorithm shows that SH-SY5Y cell lines were differentiated toward developmental stages 5–8, which is beyond the stages achieved using established protocols.^15, 16^ In addition, we observed preservation of modules highly relevant for ASD, that is, glutamatergic and GABAergic pathways. However, neither the improved cell model nor cells derived from the original protocols show preservation of modules associated with synaptic transmission. In line with current discussions about neuronal differentiation methods,^38^ we propose that protocols applied to neuronal *in vitro* models need to be evaluated in more detail.

Most differentiation protocols for SH-SY5Y cells including either RA-only or sequential treatment with RA and BDNF report a dopaminergic phenotype. However, the continuous RA-BDNF exposure protocol used here does not result in up-regulation of the rate-limiting enzyme of dopamine, that is, the TH, and leads to a down-regulation of the dopamine transporter DAT1. A negative correlation between BDNF levels and dopamine receptors DRD2 and DRD3 expression has previously been reported in rat models for stress linking the two systems.^39^ We hypothesize that the immediate activation of the BDNF/TRK system during differentiation underlies the negative effect on the dopamine system. It is also interesting to note that the expression of tyrosine receptor kinase beta in SH-SY5Y cells is activated by RA and reaches its peaks after 5 days of exposure.^12^ Thus, in line with previous publications,^12^ we emphasize the importance of selecting differentiation agents and the temporal exposure of cells to these agents based on the specific research question related to the cell model.

In summary, it is true that SH-SY5Y cells may not allow investigating all functional aspects of neurons; the continuous exposure of SH-SY5Y cells to RA and BDNF improved differentiation and thus allows investigating genes relevant in early cortical development and exploring regulatory networks associated with psychiatric disorders.

### DEX, ICA and DTW analyses are complementary approaches to identify differentiation-related gene sets

Following our aim to fully characterize the neurodevelopment of our cell model, we applied DEX, DTW and ICA. Most of the genes identified through DTW were overlapping with DEX genes, identifying the subset of DEX genes that is regulated dynamically. In all, 70% of all genes identified through ICA were also identified in the DEX approach. This again shows the usefulness of implementing three complementary approaches^20^ to identify the whole set of genes that is implicated in neuronal development. Considering the GO-term enrichment analysis, we assume that the DTW–ICA-derived expression signature corresponds to modulatory mechanisms regulating growing structures, whereas the short-term (DEX only) regulatory pattern may imply cellular checkpoint mechanisms (for example, during cell cycle regulation).

### Regulatory mechanisms of neurodevelopment are relevant in neuropsychiatric disorders

As expected, when analyzing DEX genes, cell cycle regulators were among the down-regulated, and neuron-related or differentiation-activating genes among the up-regulated set. When testing for risk gene enrichment we need to consider the overlap of gene lists across disorders. Specifically, genes implicated in ASD and FXS showed a strong overlap. This fact may in part explain the overlapping pattern of enrichment for these gene lists. However, despite the overlap between SCZ and ASD risk genes or between SCZ and SCZ-associated synaptic pruning genes, the enrichment signature was different between the lists. Additional complexity is added by the differing lengths of the disorder-specific gene lists, which results in differential power to detect a significant enrichment. Finally, it is to be expected that module-specific gene enrichment observed for a short list is even more easily observed for a long gene list. This, however, is not the case. Therefore, we are confident that our conclusions with respect to disorder-specific risk gene enrichments are valid.

In our hypothesis-free approach, we confirmed the association of ASD-, FXS-, ID- or SCZ-implicated genes with regulation of neuronal differentiation. In contrast to the other disorders, only the ID-implicated genes were enriched among down-regulated sets, pointing to a major difference in the underlying pathomechanisms despite the genetic overlap.

Our findings confirm a previous prediction of a computational study reporting that variants associated with ASD specifically will disrupt genes that are positive regulators of biological processes.^40^ This can now further be complemented by the finding that this does not apply to variants causing ID, as these also affect negative regulators. It has to be considered here that rare deleterious *de novo* CNVs detected in ASD patients are enriched for ID-implicated genes.^5^ Thus, to understand whether disruption of negative regulators of neurodevelopment induces a specific subtype of ID even in ASD individuals, we propose testing whether loss of function mutations or gene-dosage reductions of negative or positive regulators are specific to subgroups of patients with ID or with ASD and ID.

The specific analysis of dynamically regulated gene sets further suggests that ASD- and FXS-implicated genes, but not ID and SCZ-risk genes, are indeed expressed during the full trajectory of early neuronal differentiation. The overlapping genetic enrichment of ASD- and FMRP-targeted genes in the extracellular matrix-related cluster may be driven by the overlap between the gene lists. Interestingly, in this cluster we also observed enrichment for genes of functional networks targeted by rare *de novo* CNVs in ASD and genes belonging to the synaptic M12 ASD-associated module identified in a post-mortem study.

ASD genes themselves are likely to be associated with developmental processes as confirmed in the ICA analysis here. This refines our hypothesis towards an association of ASD with promoters of developmental processes,^40^ and in addition suggests that dynamic regulators of neuronal development are more vulnerable to genetic variation specifically in ASD.

Network analysis of the neuronal transcriptome identified 20 modules. Again, we observed specific gene-set enrichment for ASD-, FXS-, ID- and SCZ-related genes. As detailed above, we exclude potential power issues because we did not observe enrichment of especially longer lists (that is, ASD_AutismKB). In addition, despite a significant overlap of the gene lists, enrichment itself was not overlapping and thus the enrichments were disorder- and module-specific. An example is the down-regulated turquoise cell cycle-related module, which was enriched for genes implicated in ID, but not for ASD-risk genes. Among these genes are, for example, the X-linked mental retardation gene *UPF3B*^41^ or the CHARGE syndrome-associated gene *CHD7* (chromodomain helicase DNA-binding protein 7)^42^. Similar to the observations of DEX genes, the FMRP-targeted genes were under-represented in this ID-gene-enriched cluster.

ASD genes, but not ID-implicated genes, were significantly enriched in the pink module, which can be labeled as protein trafficking and signaling response. Both processes have previously been associated with ASD.^43, 44^ Besides the ASD-associated trafficking-related gene Neurobeachin (*NBEA)*^45^, this module contains also other several strong ASD-associated genes, that is, Neuroligin 3 (*NGL3*)^46^ or Neurexin 1 (*NRXN1*)^47^.

Protein trafficking has already been shown to be affected by mutations in known ASD-risk genes, for example, Neuroligin 4 (*NLGN4*) mutations were reported to alter the transport of NLGN4 protein to the synaptic density.^48^ These findings add to the hypothesis that axonal pathfinding and protein transport might be pathomechanisms specific to ASD.^49^

A specific overlap between ASD and ID was observed for a module associated with dendritic development (darkgreen), supporting the assumption that the pathological overlap between ASD- and ASD-related disorders with ID might originate from dendritic dysgenesis.^50^ Among the most prominent genes in this module is Reelin (*RELN*). *RELN* has repeatedly been associated with ASD^51^ as well as with lissencephaly^52^ and mental retardation.^53^ Another interesting candidate gene is the mitogen-activated protein kinase 3 (*MAPK3/ERK3*). This effector kinase mediates glutamatergic signaling in excitatory synapses, a process highly discussed in ASD etiological research.^54^

Our results support the assumption that an overlapping pathomechanism between SCZ and ASD could be affecting transcriptional responses to stress and cell morphological processes. One of the most discussed transcriptional regulators in ASD, chromodomain helicase 8 (*CDH8*), is part of the royalblue, cell morphological module. Indeed, risk variants for *CHD8* have been described for both ASD and SCZ.^30, 55^

### ASD-risk genes are modulatory hubs during neuronal differentiation

To understand the broader functional impact of gene disruptions at a system's level, the network position of the respective genes needs to be considered.^56^ We have previously shown that a rare ASD variant can alter regulatory networks of oxidative stress, energy metabolism and translation.^57^ Here, we show that risk genes for ASD are likely to be modulatory hubs central in information transfer during neuronal differentiation and thus confirm that hubs are more likely to be affected by genetic risk variants for ASD.^58^ Interestingly, ASD genes, but not ID-implicated genes, were likely to be within the shortest paths between genes (information transfer hubs), whereas ID genes but not ASD genes were among the highly connected genes. This general observation is in line with the genetic model strongly discussed in ASD research,^59, 60^ where variants causing monogenetic disorders show a high penetrance (that is, potentially affect central regulators) and variants involved in complex disorders show low penetrance (that is, modulatory variants).^61^ We thus suggest that rare highly penetrant variants affect genes of high connectivity disrupting larger parts of the network leading to a more severe phenotype such as ID, whereas variants affecting informational regulators can be bypassed and thus are of low penetrance such as in ASD without ID. This supports a recent publication reporting that common variants explain more of the ASD risk than rare variants.^62^ We hypothesize that a mutation can cause ID in ASD depending on the network position of the gene within a regulatory network. Interestingly, the observed enrichment for gene-disrupting mutations in ASD cohorts was driven by low IQ individuals.^29^ Thus, in complex disorders, the network position of an affected gene is a central parameter when estimating the pathological effect of a variant.

## Conclusion

Our work takes an unbiased view on an optimized SH-SY5Y neuronal differentiation model. Although these cell lines do not model neurodevelopment as well as organoids, iPSC or primary progenitor cultures,^63, 64^ transcriptome analysis indicates that when optimized they do capture early cortical development with high fidelity. Further, they have the advantages of ease and low cost; therefore, in some circumstances, especially molecular and drug screening, they provide a valuable model system. The profound translational analysis of the developmental trajectory showed that the genetic networks differ across related disorders and thus encourages future genetic studies that integrate genetic network structures to stratify neuropsychiatric patients for personalised interventions.
