# Supplementary material for: Transcriptomic signatures of neuronal differentiation and their association with risk genes for autism spectrum and related neuropsychiatric disorders
Source: Transl Psychiatry. 2016 Aug 2;6(8):e864–. doi: 10.1038/tp.2016.119 (PMC5022076; doi:10.1038/tp.2016.119)
Supplement: Supplementary Methods, Table 1 and Figures 1–7 [file tp2016119x1.docx]

**Supplementary Information**

**Transcriptomic signatures of neuronal differentiation and**

**their association with risk genes for autism spectrum and**

**related neuropsychiatric disorders**

Andreas G. Chiocchetti^1*^, Denise Haslinger^1^, Jason L. Stein^2^, Luis de la Torre-Ubieta^2^, Enrico Cocchi^3^, Silvia Lindlar^1^, Regina Waltes^1^, Simone Fulda^4^, Daniel H. Geschwind^2^, Christine M. Freitag^1^

1) Department of Child and Adolescent Psychiatry, Psychosomatics and Psychotherapy, JW Goethe University Frankfurt, 60528 Frankfurt am Main, Germany.

2) Neurogenetics Program, Department of Neurology, Center for Autism Research and Treatment, Semel Institute, David Geffen School of Medicine, University of California, Los Angeles, Los Angeles, CA 90095, USA.

3) Department of Biomedical and NeuroMotor Sciences, University of Bologna, 40123 Bologna, Italy

3) Institute of Experimental Cancer Research in Pediatrics, 60528 Frankfurt am Main, Germany.

**Index**

[Supplementary Data 3](#_Toc442103022)

[Supplementary Methods 3](#_Toc442103023)

[Real-time RT-PCR: 3](#_Toc442103024)

[Western Blot analysis: 3](#_Toc442103025)

[Computational analysis: 4](#_Toc442103026)

[Supplementary Tables 6](#_Toc442103027)

[Supplementary Table 1: Primer and probes (universal probe library; Roche) used in this study 6](#_Toc442103028)

[Supplementary Tables 2 to 9 are provided as separate Excel sheets. 6](#_Toc442103029)

[Supplementary Figures 7](#_Toc442103030)

[Supplementary Figure 1: Validation of neuronal differentiation and microarray data 7](#_Toc442103031)

[Supplementary Figure 2: Expression of neuronal markers 8](#_Toc442103032)

[Supplementary Figure 3: Sample correlation and principal component analysis 9](#_Toc442103033)

[Supplementary Figure 4: DTW gene-selection 9](#_Toc442103034)

[Supplementary Figure 5: pICA gene-loads 10](#_Toc442103035)

[Supplementary Figure 6: WGCNA threshold selection 11](#_Toc442103036)

[Supplementary Figure 7: Overlap of analyzed lists of risk-genes 12](#_Toc442103037)

## Supplementary Data

Expression data has been submitted to GEO (GSE69838).

## Supplementary Methods

### Real-time RT-PCR:

RNA was extracted using the GeneJet RNA purification kit and transcribed into cDNA using the RevertAid H Minus cDNA Synthesis Kit according to the manufacturer’s protocol (both Fermentas). Quantitative real-time PCR was performed using the StepOnePlus Real-Time PCR system (Life Technologies) making use of the Universal Probe Library system (UPL; Roche). One PCR reaction contained 10ng cDNA template, 1x ABsolute qPCR ROX Mix (Thermo Scientific), 200nM Primer fwd, 200nM primer rev, 100nM UPL probe and PCR-grade H_2_O. For details on primer and probe combinations see Supplementary Table 1.

### Western Blot analysis:

For protein extraction SH-SY5Y cell pellets were resuspended in 200µl NET2 buffer (20mM Tris pH 7.5, 150mM NaCl, 0.05% IGEPAL) supplemented with proteinase inhibitor (PIC Mini Complete; Roche). After a 15’ incubation on ice, the cells underwent 5 repetitive freeze (-80°C) and thaw cycles. Cell debris was centrifuged 15’ at 4°C at 17,000g. Proteins were precipitated with 1x volume 20% TCA (Roth) and washed three times for 10’ with 1ml of 100% EtOH on ice. Proteins were pelleted for 10’ at 4°C and 20,000g. Finally, air dried pellets were resuspended in 1x SDS-sample buffer (4x SDS sample buffer; 0.0627M Tris, 1% SDS, 10% Glycerol, 2% 2-Mercaptoethanol and 0.1% Bromphenol-blue).

15µg protein extract of each time point (0 to 11 days in vitro) were electrophoresed on a 12% SDS gel (10x running buffer: 250mM Tris, 1.92M Glycine, 1% SDS). The gels were blotted in a semi dry approach on a PVDF membrane for 1h applying 20V (anode buffer 1: 20%MeOH, 300mM Tris ad 1L with ddH_2_O; anode buffer2: 20%ml MeOH, 25mM Tris). Membranes were blocked for 1h with SuperBlock Blocking Buffer (Thermo Scientific). Primary antibodies were incubated overnight in blocking buffer. Secondary antibodies were incubated 1h at RT. Blots were washed in between three times for 5’ in PBS + 0.05% Tween-20. Protein size and antibody specificity allowed applying CDC2 and MAPT antibodies simultaneously (1:500 in SuperBlock; both rabbit; GeneTex). GAPDH antibody (1:2,500 in SuperBlock; mouse; Santa Cruz) was applied after stripping of blots with 100mM β-Mercaptoethanol, 2%SDS, 62.5mM Tris HCl pH 6.7 for 30’ at 50°C. Secondary antibodies (2^nd^ goat anti-rabbit poly HRP or 2^nd^ goat anti-mouse poly HRP; 1:5,000 in SuperBlock; Pierce/Thermo Scientific) were visualized using the ECL Prime Western Blot Detection Reagent (GE Healthcare).

### Computational analysis:

*Dynamic time warping analysis:*

DTW analysis was performed using the ‘*dtw*’ package in R. All expressed genes were considered for analysis (N=11,392) and compared to a noise matrix.

The noise matrix was a randomly (uniform distribution) generated time-series based on +/-1 SD of the mean expression of each gene at time-point 0h. DTW analysis was performed 100 times against 100 random noise matrices. Average DTW distance over all 100 iterations was used for further analysis. As DTW distance increases linearly with increasing noise ^1^ we defined a linear model based on the DTW distance predicting the rank of the DTW distance for each gene (R²=0.985, Supplementary Figure 2). The intercept of this regression model was interpreted as the maximum DTW distance to be observed from noise background in our cell line model.

*Parallel independent component analysis:*

Expression levels of genes within a biological system can be assumed to be a linear combination of expression patterns driven by biological functions. Independent component analysis is able to extract (statistically) independent sources that can be interpreted as potential regulatory ^2^ pathways. We included all genes that were expressed (N=11,392) above background. The gene-load or eigenvalue (first component) of a gene within such an independent source is thus a measure for the contribution to it. Among the 250 iterations performed component assignment and direction of gene loads is random. To match the individual extracted components across all iterations we used Pearson-correlation of the absolute gene-load values.

*Weighted gene co-expression network analysis:*

The signed adjacency is defined as a=(0.5*(1+cor))^stp, where stp is the soft thresholding power chosen to obtain a scale free network. Here, a stp of 32 resulted in a scale free network based on a regression coefficient R²>0.8 from a scale free topology modeling approach. Modules (i.e. genes with high topological overlap) were defined using hierarchical clustering (distance=distance of topological overlap, method=average linkage) and the ‘cutreeDynamic’ algorithm as published, with a minimum module size of N=30 ^3^. Modules were clustered (distance=Euclidean, linkage=complete) based on correlation distance between their Eigenvalues (Eigengene values) and merged below a cluster height=0.1.

## Supplementary Tables

### Supplementary Table 1: Primer and probes (universal probe library; Roche) used in this study

| **Gene** | **Forward** | **Reverse** | **UPL probe** |
| --- | --- | --- | --- |
| *CDK1* | tggatctgaagaaatacttggattcta | caatcccctgtaggatttgg | 79 |
| *CNTNAP2* | cctggagctctacagccagt | tcaccccattcatcctcaa | 26 |
| *DHCR7* | gccatggtcaagggctac | ttgtaaaagaaattgcctgtgaat | 60 |
| *DRD4* | gctcttcgtctactccgaggt | cgcacaggttgaagatgga | 66 |
| *GABRB3* | tgagctcccgcagttctc | cagtgacagtcgaggataggc | 54 |
| *GAPDH* | agccacatcgctcagacac | gcccaatacgaccaaatcc | 60 |
| *GRIA2* | actgacaccccacatcgac | tcgaaaactgggagcagaaa | 10 |
| *GRIK2* | tggatattctcaaggaaccacac | tcacagcaaatctgaatgcaa | 73 |
| *GRIN1* | ccgcatgtccatctactcg | tggactggtgggagtaggg | 4 |
| *GRM1* | atgtctctgcagtccacacg | cagaatgggcgatacagagg | 44 |
| *GRM4* | tgaccacctgcaccttagaa | actgtcttcttccgctcacc | 74 |
| *MAPT* | accacagccaccttctcct | cagccatcctggttcaaagt | 55 |
| *NRXN1* | ccggagccaggaacttaga | tggtaaggatttgtatgtttctttagc | 63 |
| *POLR2F* | gatgaagggctagatgacttgg | ccagaggggaggatctcg | 2 |
| *SHANK3* | cacggaccaagtctgtaggg | gtcttgcatcgaggtgctc | 11 |
| *TH* | cctggtcaccaagttcgac | gtacacctggtccgagaagc | 85 |

### Supplementary Tables 2 to 9 are provided as separate Excel sheets.

## Supplementary Figures


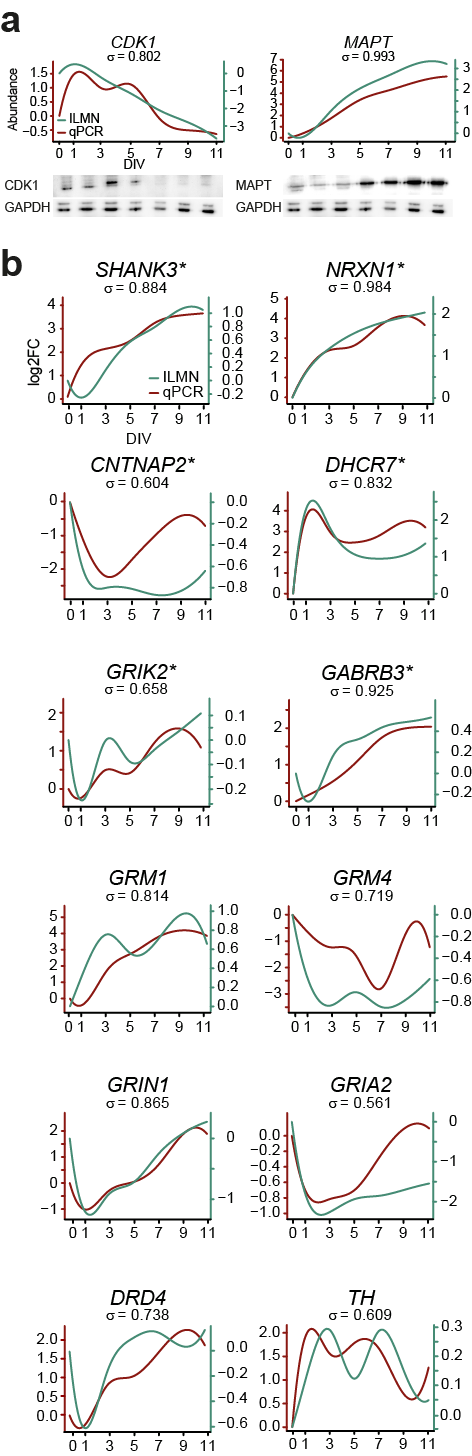


Supplementary Figure 1: Validation of neuronal differentiation and microarray data

(**a**) Neuronal differentiation was confirmed at mRNA (top) and protein level (bottom) by decreased expression of cell division marker CDK1 and increased expression of neuronal marker MAPT. Gene expression (log2 fold-change; log2FC) normalized to time-point 0 was analyzed using Illumina HumanHT-12 v4 Expression BeadChips (ILMN, turquoise) and real-time RT-PCR (qPCR, red). Western-blot images are all from the same blot (reference protein: GAPDH). (**b**) Validation of microarray (turquoise) using real-time RT-PCR (red). log2FC normalized to time-point 0 of selected ASD-risk genes (asterisk) and neuronal receptors are shown.


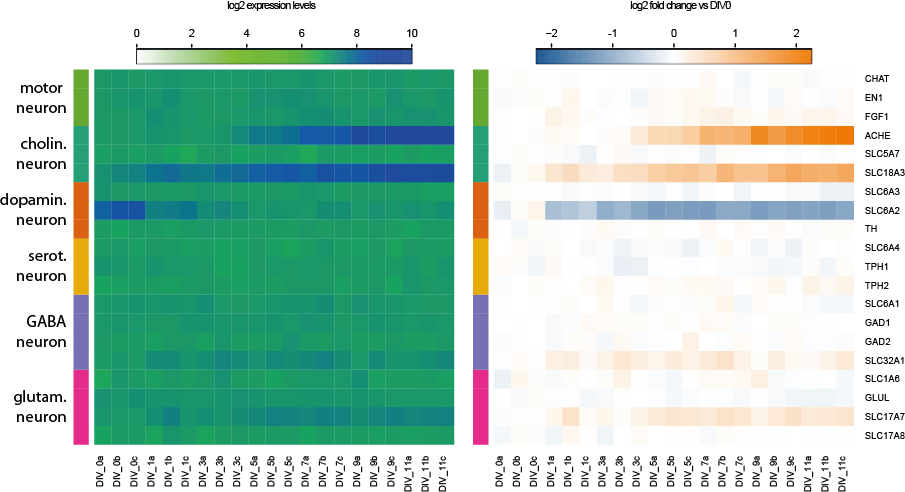


### Supplementary Figure 2: Expression of neuronal markers

Expression of typical neuronal markers for motor neurons, cholinergic, dopaminergic, serotonergic, GABAergic and glutamatergic markers are shown as log2 expression values (left panel) and as log2 fold change compared to undifferentiated cells (right panel). Up-regulation of cholinergic markers acetylcholinesterase *(ACHE)* and vesicular acetylcholine transporter *(SLC18A3)* as well as a mild up-regulation of GABA vesicular transporter *(SLC32A1)* and vesicular glutamate transporter 1 *(SLC17A7)* was observed. Interestingly, a down-regulation of the dopaminergic transporter *SLC6A2* was observed in our cell model.

**
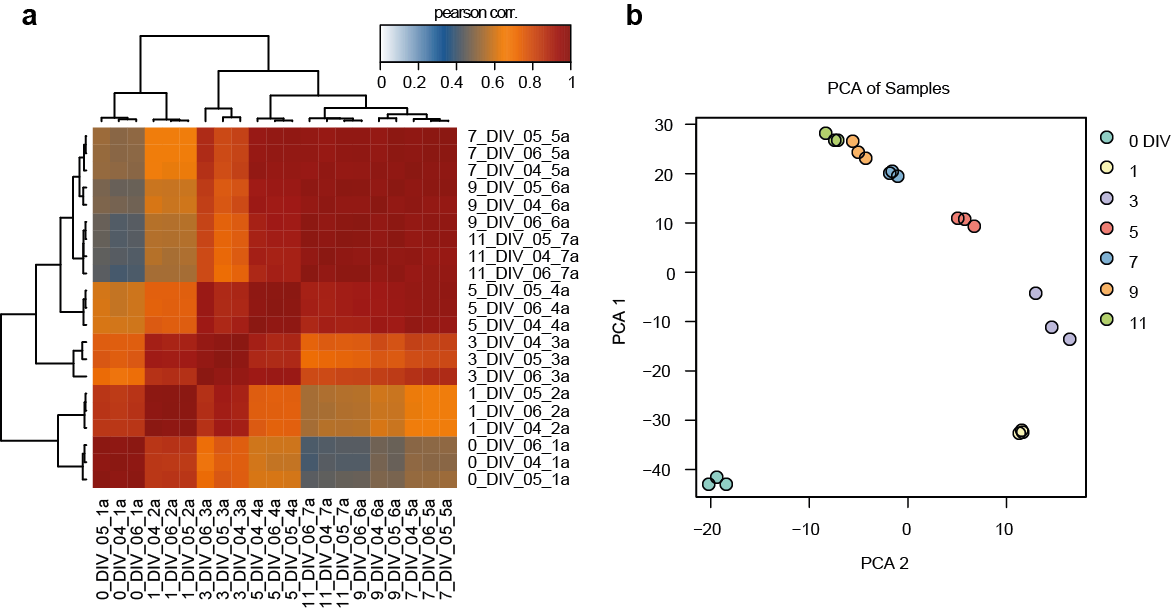
**

### Supplementary Figure 3: Sample correlation and principal component analysis

(**a**) Top 2000 genes with the largest variance across all samples were used for sample correlation analysis. Hierarchical clusters (complete linkage) are based on 1 – Pearson correlations distance measures. No outlier samples were detected and replicates clustered perfectly together. Samples of later differentiation stages 7-11 are highly correlated suggesting that most changes in expression occur during the first week (**b**) Principle component analysis based on all expressed genes again showed stable signatures across replicates. First two components are plotted.


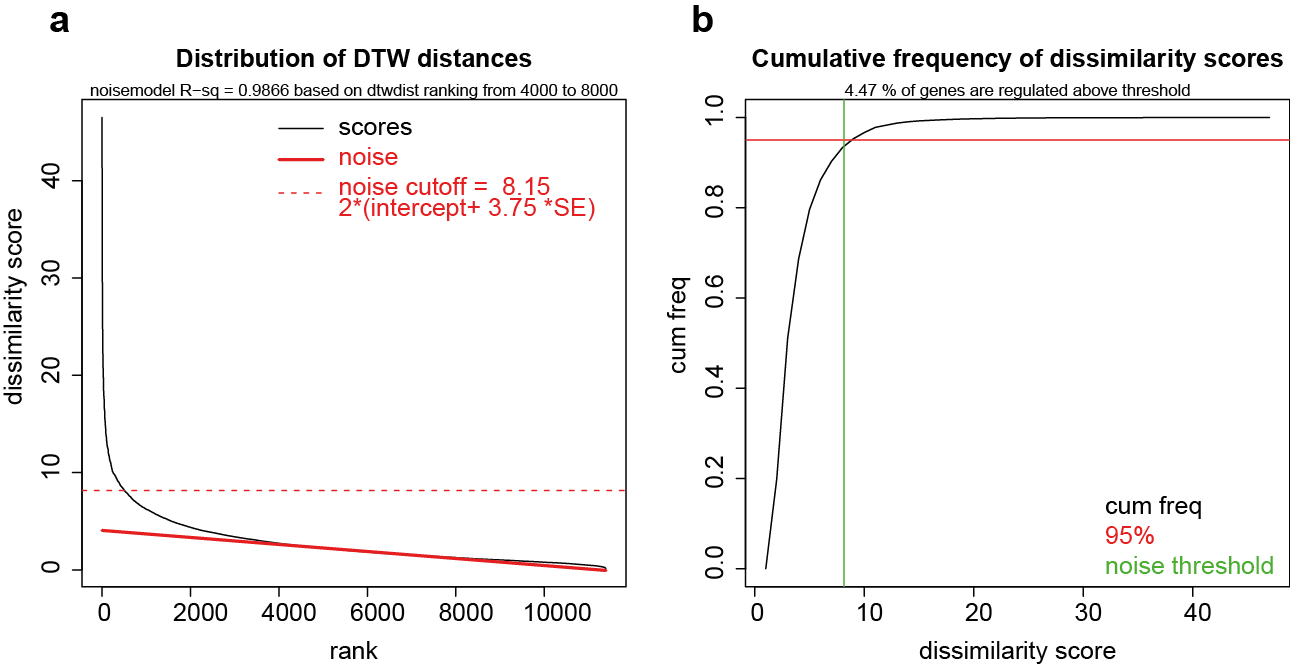


### Supplementary Figure 4: DTW gene-selection

(**a**) DTW distance plotted by rank. Noise model was calculated by regressing the dissimilarity score via the genes ranking 4,000-8,000. The intercept of the model was considered as the maximum DTW distance to be observed through noise. The cut-off was defined as 2x the upper 99% confidence interval of the intercept. (**b**) Cumulative frequency plot of DTW distance scores shows that 4.3% (N=509) genes where above the cut-off.


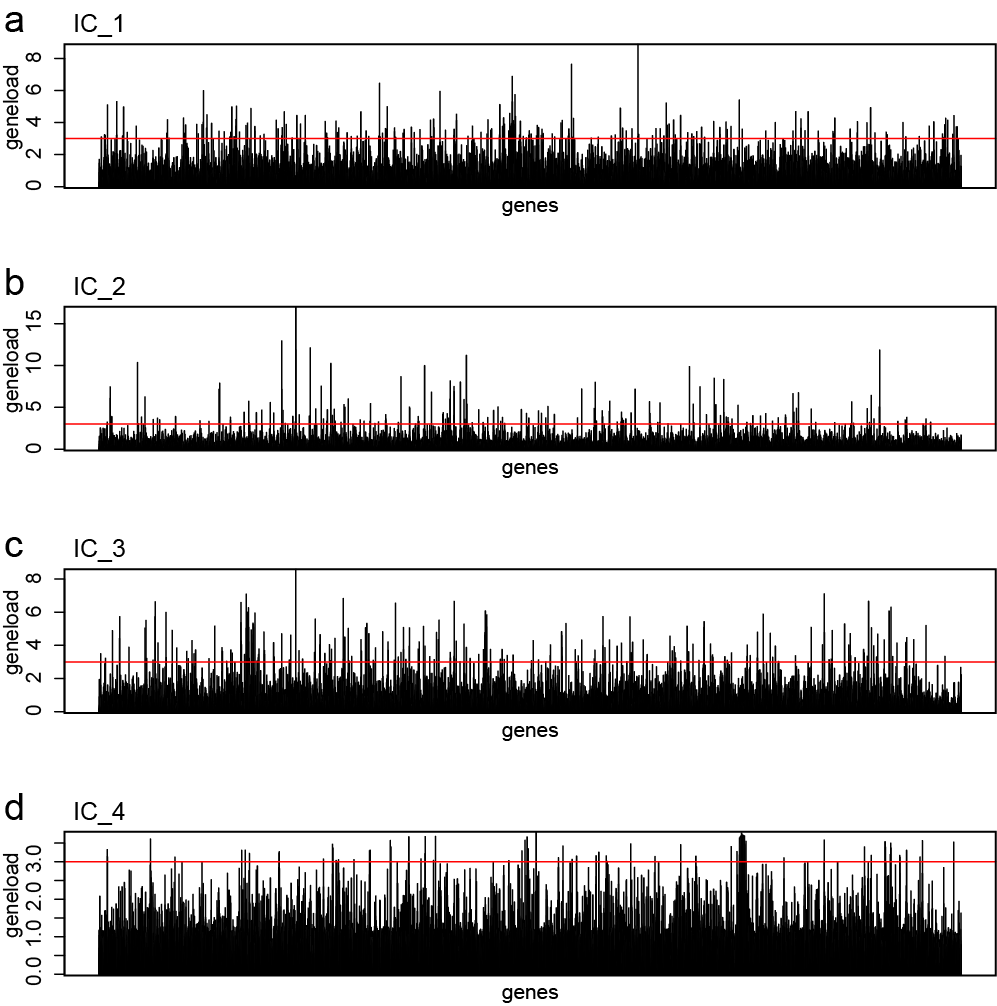


### Supplementary Figure 5: pICA gene-loads

Absolute values of genes loading onto the four extracted components. Genes with loads above 3 were considered as major drivers within this component.


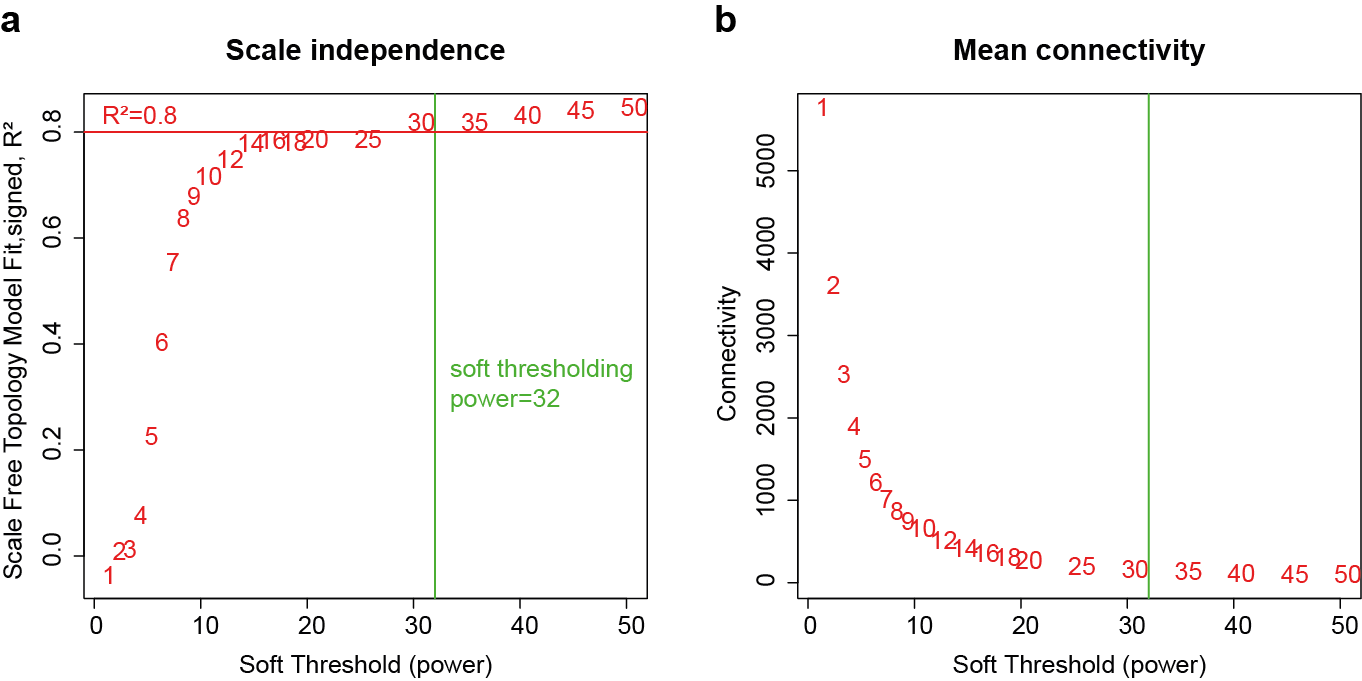


### Supplementary Figure 6: WGCNA threshold selection

We chose to select a threshold for WGCNA signed network analysis such as a scale free network can be assumed based on the scale-free model regression coefficient of R²>0.8 as suggested ^3^. (**a**) Using all genes expressed in the SH-SY5Y model a power of 32 fulfilled the R²>0.8 criterion. (**b**) Mean connectivity of resulting network based on adjacency measures for tested thresholds.


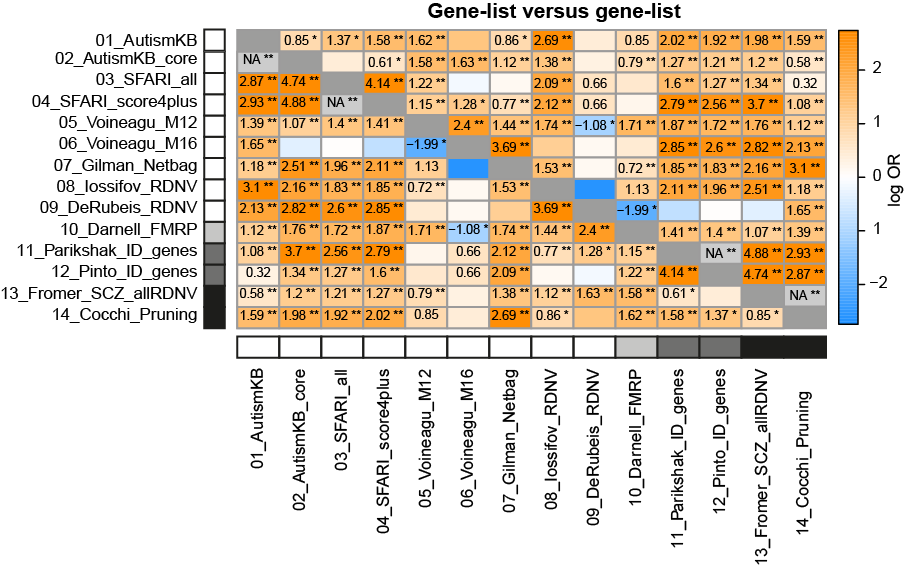


### Supplementary Figure 7: Overlap of analyzed lists of risk-genes

Log odds ratios with false discovery rate FDR>0.1 of Fisher’s exact test comparing individual gene-lists are shown. Asterisks mark FDR<0.05*, FDR<0.01**. List 02_AutismKB_core is a subset of 01_AutismKB. The same applies to 04_SFARI_score4plus and 03_SFARI_all. Gene lists 05_Voineagu_M12 and 06_Voineagu_M16 are mutually exclusive by definition. Grey shaded boxes mark lists of genes implicated in ASD (white), FXS (light grey), ID (dark grey) and SCZ (black). The set of all genes targeted on the array was used as reference genome.

**References**

1 Wexler EM, Rosen E, Lu D, Osborn GE, Martin E, Raybould H *et al.* Genome-wide analysis of a Wnt1-regulated transcriptional network implicates neurodegenerative pathways. *Sci Signal* 2011; **4**: ra65.

2 Chiappetta P, Roubaud MC, Torrésani B. Blind source separation and the analysis of microarray data. *J. Comput. Biol.* 2004; **11**: 1090–1109.

3 Langfelder P, Horvath S. WGCNA: an R package for weighted correlation network analysis. *BMC Bioinformatics* 2008; **9**: 559.
